# Nonconvulsive status epilepticus manifesting as rapidly progressive dementia and infarction in the splenium of the corpus callosum: A case report

**DOI:** 10.1097/MD.0000000000025263

**Published:** 2021-04-16

**Authors:** Qian Zhao, Lichao Sun, Boqi Hu, Weihong Lin

**Affiliations:** a Department of Neurology, Neuroscience Center; b Department of Emergency, The First Hospital of Jilin University; c Department of Radiology, China-Japan Friendship Hospital of Jilin University, Changchun, Jilin, China.

**Keywords:** case report, infarction, nonconvulsive status epilepticus, rapidly progressive dementia, splenium of the corpus callosum

## Abstract

**Rationale:**

Nonconvulsive status epilepticus (NCSE) is a heterogeneous disease with multiple subtypes. NCSE poses great diagnostic and therapeutic challenges due to the lack of typical symptoms. Here, we report a case of NCSE manifesting as rapidly progressive dementia (RPD) and infarction in the splenium of the corpus callosum. Additionally, the relevant literature was reviewed.

**Patient concerns:**

A 63-year-old man presented with RPD. Electroencephalogram (EEG) revealed NCSE, and brain magnetic resonance imaging (MRI) showed an isolated infarction in the splenium of the corpus callosum. Mini-mental state examination showed moderate cognitive impairment (14/30 points).

**Diagnosis:**

A diagnosis of NCSE with RPD and infarction in the splenium of the corpus callosum was made.

**Interventions:**

The patient was treated with intravenous diazepam (10 mg), oral levetiracetam (1.0g twice daily), oral sodium valproate (0.2g twice daily), and intramuscular phenobarbital sodium (0.2g once daily).

**Outcomes:**

After the treatment, the symptoms were improved. The patient could answer questions. Repeated EEG showed that the background a rhythm was slightly overdeveloped, and no clinical or electrical seizures were observed. After discharge, the patient was treated with oral levetiracetam (1.0g twice daily) and oral sodium valproate (0.2g twice daily) for 6 months. At the last follow-up, the patient had clear consciousness, sensitive response, and fluent answering ability. Repeated mini-mental state examination showed that his cognitive function was significantly improved (28/30 points); nevertheless, the lesion in the splenium of corpus callosum remained unchanged on MRI.

**Lessons:**

NCSE manifesting as RPD and infarction in the splenium of the corpus callosum is extremely rare. Epileptic events and focal infarction are usually overlooked in patients with dementia, and the diagnostic value of MRI and EEG should be highlighted

## 1 Introduction

Nonconvulsive status epilepticus (NCSE) refers to a distinct subtype of status epilepticus with no obvious tonic-clonic activity.^[1]^ This condition is clinically latent, and there are no typical onset symptoms. NCSE often occurs following a coma or post-epileptic state, and it is usually underdiagnosed.^[2]^ Although there have been numerous reports of NCSE secondary to stroke, NCSE complicated with infarction in the splenium of the corpus callosum is exceedingly rare because of the rich blood supply of the corpus callosum.^[3]^ Rapidly progressive dementia (RPD) refers to dementia that progresses subacutely, typically over the course of weeks to months. RPD is caused by vascular occlusion in the corpus callosum or thalamus or multiple cerebral infarctions, and some of RPD cases are reversible and curable.^[4]^ According to the literature, epilepsy with higher brain dysfunction is associated with dementia; for example, temporal lobe epilepsy can lead to memory loss or behavioral abnormalities, which is similar to Alzheimer disease or frontotemporal dementia. Epilepsy with higher brain dysfunction can be classified into a transient subtype and a persistent subtype, and the latter form is caused by NCSE or antiepileptic drug-responsive neurofunctional impairment.^[5]^ Currently, NCSE poses great diagnostic and therapeutic challenges due to the lack of typical symptoms. In the present report, we present a case of NCSE manifesting as RPD and irreversible infarction in the splenium of the corpus callosum and review the relevant literature.

## 2 Case report

**Figure 1:**
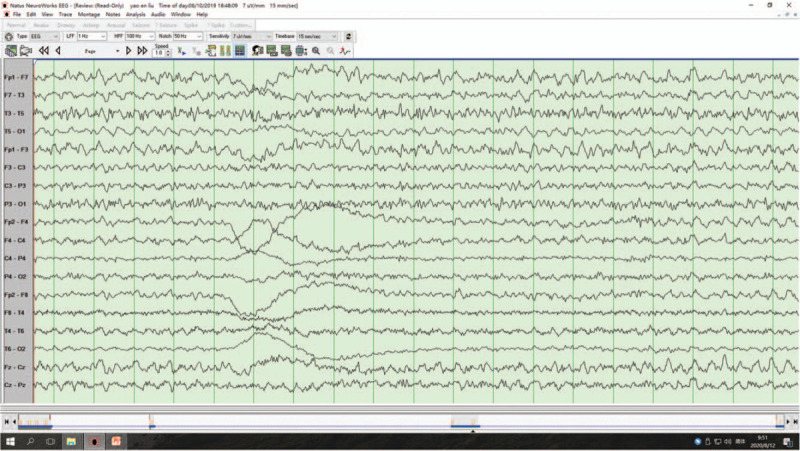
Electroencephalogram on admission. Electroencephalogram monitored spike and spike-slow waves (4–6 Hz) originating from the left temporal area (F7/T3/T1) during the ictal period (lasting for 6–30 s).

**Figure 2:**
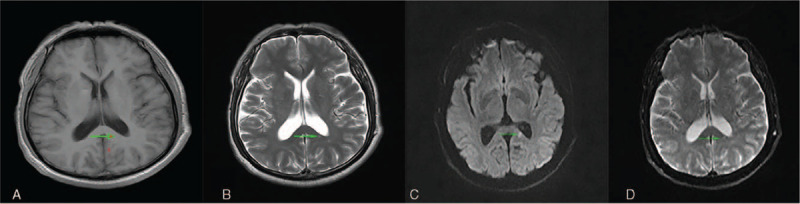
Brain magnetic resonance imaging on admission. Brain magnetic resonance imaging showed an isolated lesion in the splenium of the corpus callosum with hypointensity on T1-weighted imaging (A) and the apparent diffusion coefficient map (F) and hyperintensity on T2-weighted imaging (B), and diffusion-weighted imaging (C).

**Figure 3:**
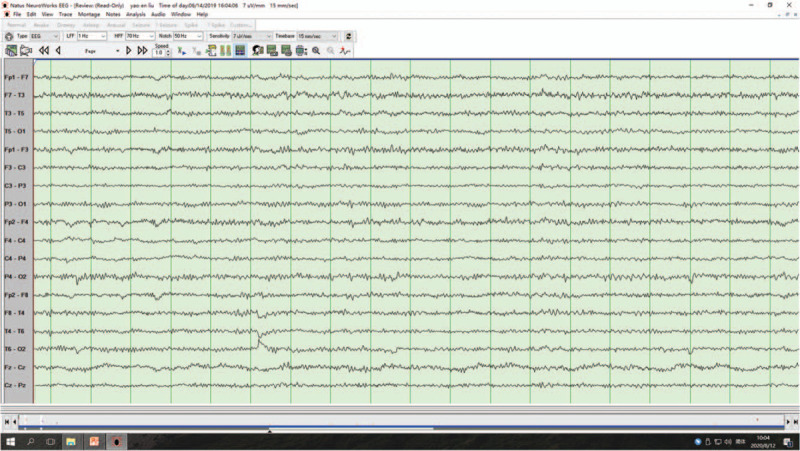
Electroencephalogram after treatment. Electroencephalogram after treatment showed the background α rhythm was slightly overdeveloped, but no clinical or electrical seizures were observed.

**Figure 4:**
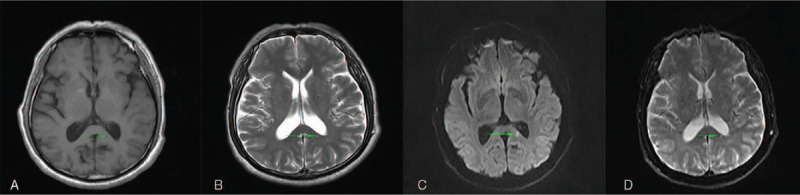
Brain magnetic resonance imaging after a 6-mo follow-up. Brain magnetic resonance imaging showed an isolated lesion in the splenium of the corpus callosum with hypointensity on T1-weighted imaging (A) and the apparent diffusion coefficient map (D) and hyperintensity on T2-weighted imaging (B), diffusion-weighted imaging (C).

A 63-year-old man presented to us with RPD. Twenty days previously, the patient developed lags in response. However, other behaviors were normal, and he was able to take care of himself. Five days before admission, the symptom was aggravated, and the intermittent attacks were frequent (4–5 episodes per day; each episode lasted for approximately 5 minutes). The patient could not recognize common objects or answer questions accurately. He was completely normal during the interictal period. Brain computed tomography showed no remarkable abnormalities. During the previous 5 days, the patient developed severe paroxysmal headache (7–8 episodes per day). Physical examination revealed fuzzy consciousness and slow response. There was no dysarthria, cranial nerve paresis, or motor function disturbance, and bilateral pathological signs were negative. Cerebrospinal fluid examination after lumbar puncture showed a slightly increased protein level (0.93 g/dL; normal range, 0.15–0.45 g/dL), an increased glucose level (4.8 mmol/L; normal range, 2.3–4.1 mmol/L), and an elevated leukocyte count (9 × 10^6^/L; normal range, (0–8) × 10^6^/L). Antibodies against autoimmune encephalitis and 1433 protein and paraneoplastic antibodies were negative. Mini-mental state examination showed moderate cognitive impairment (14/30 points). Electroencephalogram (EEG) monitored spike and spike-slow waves (4–6 Hz) originating from the left temporal area (F7/T3/T1) during the ictal period (lasting for 6–30 seconds; Fig. 1). Brain magnetic resonance imaging (MRI) showed an isolated lesion in the splenium of the corpus callosum with hyperintensity on diffusion-weighted imaging and hypointensity on the apparent diffusion coefficient map (Fig. 2). A diagnosis of NCSE with RPD and infarction in the splenium of the corpus callosum was made. The patient was treated with intravenous diazepam (10 mg), oral levetiracetam (1.0 g twice daily), oral sodium valproate (0.2 g twice daily), and intramuscular phenobarbital sodium (0.2 g once daily). After the treatment, the symptoms were improved. The patient could answer questions. Repeated EEG showed that the background α rhythm was slightly overdeveloped, and no clinical or electrical seizures were observed (Fig. 3). After discharge, the patient was treated with oral levetiracetam (1.0 g twice daily) and oral sodium valproate (0.2 g twice daily) for 6 months. At the last follow-up, the patient had clear consciousness, sensitive response, and fluent answering ability. Repeated mini-mental state examination showed that his cognitive function was significantly improved (28/30 points); nevertheless, the lesion in the splenium of corpus callosum remained unchanged on MRI (Fig. 4).

Reporting of this case was approved by the Ethics Committee of The First Hospital of Jilin University. Written informed consent was obtained from the patient's relatives.

## 3 Discussion

Clinical manifestations of NCSE are variable, with consciousness changes being the most common, and other symptoms include speech disturbances, myoclonic, behavioral abnormalities, anxiety, delirium, extrapyramidal symptoms, and hallucinations. In 2007, Kaplan proposed the EEG diagnostic criteria for adult NCSE.^[6]^ In addition to repeated extensive or focal spike waves, polyspike waves, sharp waves, spike-slow waves, and sharp-slow waves, EEG characteristics of NCSE should include:

(1) rhythmic (θ-δ) waves with a frequency ≥0.50 Hz and alterations in amplitude and/or frequency,
(2) a pattern change with a frequency change >1 Hz or position change, and
(3) background waves with a decreased amplitude and/or frequency following periodic discharges.^[6]^

EEG monitoring is the gold standard for the diagnosis of NCSE.^[7]^

Status epilepticus is usually underdiagnosed, especially in patients without convulsive episodes. When patients with NCSE are admitted to the emergency room, EEG is usually not available. For these cases, experimental usage of intravenous benzodiazepines may facilitate the diagnosis. Notably, the cognitive symptoms of NCSE may be improved several months after the administration of benzodiazepines.^[8]^ In the current case, the patient developed lags in response 20 days before admission, and then the symptom was aggravated. The clinical manifestation and EEG evidence supported a diagnosis of NCSE.

NCSE can be associated with stroke in the acute and chronic stages. However, the infarction in the splenium of the corpus callosum is uncommon in patients with NCSE. MRI is the optimal modality for the diagnosis of corpus callosum infarction. As previously reported, the incidence of acute corpus callosum infarction is 2.3%, and the negative rate of computed tomography examination was 76.4% even 24 hours after onset.^[9]^ The corpus callosum, located at the base of the longitudinal fissure of the brain, is the largest connective fibers in the central nervous system and connects the neocortex in the bilateral hemispheres. The splenium of the corpus callosum is the posterior part of the corpus callosum, which connects the temporal and occipital lobes. In the previous literature, the infarction often occurs in the genu or body of the corpus callosum.^[10]^ David et al reported 5 patients with corpus callosum infarction. They found the infarction only involved the genu or body of the corpus callosum; none involved the splenium of the corpus callosum. They hypothesized that this phenomenon might be due to the difference in the patient population.^[11]^ Corpus callosum infarction is caused by hemodynamic changes secondary to cerebral arteriosclerosis, and clinical manifestations mainly include speech disorders and cognitive impairment. The subcallosal artery supplies the beak and genu of the corpus callosum as well as the anterior fornix, and its occlusion causes acute loss of short-term memory, which is known as “amnestic syndrome of the subcallosal artery” and appear “cup sign” on MRI.^[12,13]^ Rabinstein et al reported a case of RPD caused by bilateral internal carotid artery occlusion with infarction of the whole corpus callosum.^[14]^ These findings support that RPD may be caused by occlusion of large vessels in the thalamus and corpus callosum. Zhang et al reported 2 cases of infarction in the splenium of the corpus callosum; both patients presented with sudden cognitive impairment and sensorimotor dysfunctions in the right limb.^[15]^ Notably, in the above studies, no NCSE was observed, and the reversibility of the infarction in the splenium of the corpus callosum was not reported. In the current case, the splenium infarction was irreversible.

At present, there is no consensus on the definition and diagnostic criteria of RPD. Kelley et al believed that RPD progresses from clinical onset to severe dementia or death within 18 months.^[16]^ Geschwind et al proposed that RPD progresses to dementia within 1 to 2 years after clinical onset, typically over the course of weeks to months.^[17]^ Papageorgiou et al considered that RPD is a subtype of dementia with obvious cognitive impairment within a few months, and RPD cases with a clinical course of <3 years were included in their study.^[18]^ Meanwhile, Josephs et al found that the survival time of RPD is less than 4 years, generally 0.2 to 3.5 years.^[19]^ Geschwind et al investigated the etiology of RPD and found that 62% of all RPDs were prion diseases, 15% were neurodegenerative diseases, 8% were autoimmune diseases, 4% were infectious diseases, and 2% were other diseases (such as neoplastic, metabolic, psychiatric, and vascular diseases).^[20]^

NCSE with concomitant RPD is rarely reported. NCSE is most commonly associated with focal epileptic seizures with impaired consciousness, and one of the main clinical manifestations is an acute confusional state.^[21]^ As a potential cause of consciousness changes in the elderly, NCSE should be included in the differential diagnoses for patients with rapid cognitive decline.^[22]^ Cordonnier and Reuck hypothesized that epilepsy and new-onset dementia may be caused by changes in white matter, asymptomatic infarcts, and microbleeds, and that poststroke seizures are independent predictors of new-onset dementia.^[23,24]^ Epileptic seizures can occur in 3.6% of individuals with dementia, and it is often difficult to recognize when the clinical manifestations are only slight behavioral changes.^[25]^ In the previous literature, some epileptic patients developed reversible splenial lesion syndrome.^[26]^ However, in the current case, the infarction in the splenium of the corpus callosum was irreversible, which is different from reversible splenial lesion syndrome.

## 4 Conclusion

NCSE manifesting as RPD and infarction in the splenium of the corpus callosum is extremely rare. Clinicians should be aware of this distinct condition. Epileptic events and focal infarction are commonly overlooked in patients with dementia, and the diagnostic value of MRI and EEG should be highlighted.

## Author contributions

**Data curation:** Qian Zhao.

**Investigation:** Qian Zhao.

**Resources:** Qian Zhao.

**Writing – original draft:** Qian Zhao, Lichao Sun.

**Writing – review & editing:** Qian Zhao, Weihong Lin, Boqi Hu.

## References

[1] Hamad AP Ferrari-Marinho T Caboclo LO. Nonconvulsive status epilepticus in epileptic encephalopathies in childhood. Seizure 2020;80:212–20. 32645639 10.1016/j.seizure.2020.06.024

[2] Baker AM Yasavolian MA Arandi NR. Nonconvulsive status epilepticus: overlooked and undertreated. Emerg Med Pract 2019;21:01–24. 31557430

[3] Halalmeh DR Klinger N Azad S. Delayed cerebral ischemia of the corpus callosum: a case report. Cureus 2019;11:e6379. 31938657 10.7759/cureus.6379 PMC6957044

[4] Geschwind MD Haman A Miller BL. Rapidly progressive dementia. Neurol Clin 2007;25:783–807. 17659190 10.1016/j.ncl.2007.04.001 PMC2706263

[5] Sugimoto A Futamura A Kawamura M. Epilepsy and dementia. Brain Nerve 2012;64:1399–404. 23209066

[6] Kaplan PW. EEG criteria for nonconvulsive status epilepticus. Epilepsia 2007;48: Suppl 8: 39–41. 10.1111/j.1528-1167.2007.01345.x 18329995

[7] Sambin S Gaspard N Legros B. Role of epileptic activity in older adults with delirium, a prospective continuous EEG study. Front Neurol 2019;10:263. 30941098 10.3389/fneur.2019.00263 PMC6434717

[8] Licht EA Fujikawa DG. Nonconvulsive status epilepticus with frontal features: quantitating severity of subclinical epileptiform discharges provides a marker for treatment efficacy, recurrence and outcome. Epilepsy Res 2002;51:13–21. 12350380 10.1016/s0920-1211(02)00107-9

[9] Sun X Li J Fan C. Clinical, neuroimaging and prognostic study of 127 cases with infarction of the corpus callosum. Eur J Neurol 2019;26:1075–81. 30793437 10.1111/ene.13942 PMC6767551

[10] Li S Sun X Bai YM. Infarction of the corpus callosum: a retrospective clinical investigation. PLoS One 2015;10:e0120409. 25785450 10.1371/journal.pone.0120409 PMC4364734

[11] Kasow DL Destian S Braun C. Corpus callosum infarcts with atypical clinical and radiologic presentations. AJNR Am J Neuroradiol 2000;21:1876–80. 11110540 PMC7974283

[12] Moussouttas M Giacino J Papamitsakis N. Amnestic syndrome of the subcallosal artery: a novel infarct syndrome. Cerebrovasc Dis 2005;19:410–4. 15925871 10.1159/000086104

[13] Pardina-Vilella L Pinedo-Brochado A Vicente I. The goblet sign in the amnestic syndrome of the subcallosal artery infarct. Neurol Sci 2018;39:1463–5. 29713937 10.1007/s10072-018-3425-z

[14] Rabinstein AA Romano JG Forteza AM. Rapidly progressive dementia due to bilateral internal carotid artery occlusion with infarction of the total length of the corpus callosum. J Neuroimaging 2004;14:176–9. 15095565

[15] Zhang J Tang Y Sun Y. Corpus callosum infarction with cognitive dysfunction: two case reports and literature review. Neuropsychiatr Dis Treat 2018;14:511–5. 29491710 10.2147/NDT.S155487 PMC5815477

[16] Kelley BJ Boeve BF Josephs KA. Rapidly progressive young-onset dementia. Cogn Behav Neurol 2009;22:22–7. 19372767 10.1097/WNN.0b013e318192cc8d PMC2769010

[17] Geschwind MD. Rapidly progressive dementia: prion diseases and other rapid dementias. Continuum (Minneap Minn) 2010;16:31–56. 22810280 10.1212/01.CON.0000368211.79211.4c

[18] Papageorgiou SG Kontaxis T Bonakis A. Rapidly progressive dementia: causes found in a Greek tertiary referral center in Athens. Alzheimer Dis Assoc Disord 2009;23:337–46. 19561440 10.1097/WAD.0b013e31819e099b

[19] Josephs KA Ahlskog JE Parisi JE. Rapidly progressive neurodegenerative dementias. Arch Neurol 2009;66:201–7. 19204156 10.1001/archneurol.2008.534 PMC2764283

[20] Geschwind MD Shu H Haman A. Rapidly progressive dementia. Ann Neurol 2008;64:97–108. 18668637 10.1002/ana.21430 PMC2647859

[21] Dupont S Kinugawa K. Nonconvulsive status epilepticus in the elderly. Rev Neurol (Paris) 2020;176:701–9. 32169326 10.1016/j.neurol.2019.12.007

[22] Chiara C Giovanni A Giovanni P. Nonconvulsive seizures and dementia: a case report. Int J Alzheimers Dis 2011;2011:690305. 21559184 10.4061/2011/690305 PMC3089911

[23] Cordonnier C Hénon H Derambure P. Early epileptic seizures after stroke are associated with increased risk of new-onset dementia. J Neurol Neurosurg Psychiatry 2007;78:514–6. 17435186 10.1136/jnnp.2006.105080 PMC2117834

[24] De Reuck J De Clerck M Van Maele G. Vascular cognitive impairment in patients with late-onset seizures after an ischemic stroke. Clin Neurol Neurosurg 2006;108:632–7. 16316720 10.1016/j.clineuro.2005.10.008

[25] Rao SC Dove G Cascino GD. Recurrent seizures in patients with dementia: frequency, seizure types, and treatment outcome. Epilepsy Behav 2009;14:118–20. 18782632 10.1016/j.yebeh.2008.08.012 PMC2875670

[26] Piri Cinar B Akar H Taylan A. A rare cause of reversible splenial lesion syndrome: a case report with epilepsy. Balkan Med J 2018;35:122–3. 28958981 10.4274/balkanmedj.2017.0733 PMC5820443

